# Coastal Bacterioplankton Community Dynamics in Response to a Natural Disturbance

**DOI:** 10.1371/journal.pone.0056207

**Published:** 2013-02-07

**Authors:** Sara K. Yeo, Megan J. Huggett, Alexander Eiler, Michael S. Rappé

**Affiliations:** 1 Hawaii Institute of Marine Biology, School of Ocean and Earth Science and Technology, University of Hawaii, Kaneohe, Hawaii, United States of America; 2 Department of Oceanography, School of Ocean and Earth Science and Technology, University of Hawaii, Honolulu, Hawaii, United States of America; University of Delaware, United States of America

## Abstract

In order to characterize how disturbances to microbial communities are propagated over temporal and spatial scales in aquatic environments, the dynamics of bacterial assemblages throughout a subtropical coastal embayment were investigated via SSU rRNA gene analyses over an 8-month period, which encompassed a large storm event. During non-perturbed conditions, sampling sites clustered into three groups based on their microbial community composition: an offshore oceanic group, a freshwater group, and a distinct and persistent coastal group. Significant differences in measured environmental parameters or in the bacterial community due to the storm event were found only within the coastal cluster of sampling sites, and only at 5 of 12 locations; three of these sites showed a significant response in both environmental and bacterial community characteristics. These responses were most pronounced at sites close to the shoreline. During the storm event, otherwise common bacterioplankton community members such as marine *Synechococcus* sp. and members of the SAR11 clade of *Alphaproteobacteria* decreased in relative abundance in the affected coastal zone, whereas several lineages of *Gammaproteobacteria*, *Betaproteobacteria*, and members of the Roseobacter clade of *Alphaproteobacteria* increased. The complex spatial patterns in both environmental conditions and microbial community structure related to freshwater runoff and wind convection during the perturbation event leads us to conclude that spatial heterogeneity was an important factor influencing both the dynamics and the resistance of the bacterioplankton communities to disturbances throughout this complex subtropical coastal system. This heterogeneity may play a role in facilitating a rapid rebound of regions harboring distinctly coastal bacterioplankton communities to their pre-disturbed taxonomic composition.

## Introduction

Microorganisms have long been recognized as key players in food web dynamics and biogeochemical cycling in the global ocean, due largely to bulk measures of microbial standing stocks and activity such as bacterial production and respiration [1], [2], [3]. While it is generally considered that the genetic and physiological diversity observed in marine microorganisms reflects their ability to assume diverse roles in biogeochemical cycling in the oceans, a major contemporary challenge for microbial oceanographers is to link this information with specific processes and rates. Determining the factors that structure community composition in the environment can lend valuable information to determining the ecological roles of bacterioplankton populations. In coastal environments, multiple environmental variables have been observed to co-vary with pelagic microbial community composition, including salinity, inorganic nutrient (primarily nitrogen and phosphorus) concentrations, turbidity, and the concentration of labile organic compounds (e.g. [4], [5], [6], [7]). However, a comprehensive understanding of the spatial heterogeneity of aquatic microbial communities in response to gradients in environmental conditions remains elusive. One general observation is that resident freshwater and marine planktonic microbial communities are genetically distinct, but mix along estuarine gradients in coastal systems (e.g. [4], [8], [9], [10], [11], [12], [13]). Irrespective of estuaries, coastal systems have also been observed to harbor distinct planktonic microbial assemblages [14], [15].

Conditions of strong environmental forcing frequently cause changes in physical and biogeochemical properties of aquatic systems. In coastal regions, irregular storms and heavy rainfall may introduce temporal and spatial variations by increasing freshwater runoff that alters environmental conditions and introduces allochthonous material (including microorganisms) into the system. Nutrient pulses from storms have been shown to shift a nitrogen-limited coastal system to phosphorus limitation, with relatively fast recovery times ranging from three to eight days [16], [17]. Under such conditions, it is likely that members of the microbial assemblage present during the mean ecosystem state may be replaced by organisms that are usually rare. These storm events may trigger a succession within the microbial community, until it eventually recovers and returns to its normal composition.

Kaneohe Bay on the northeastern shore of Oahu, Hawaii was chosen as a model system to study a natural perturbation event at high spatial and temporal resolution, as the region surrounding the bay is highly urbanized and experiences irregular, heavy subtropical storms. In many urbanized coastal ecosystems, anthropogenic activities such as stream channelization and dredging have severely impacted the physical and geochemical characteristics of the nearshore environment. Combined with episodic events of heavy rainfall that increase the influx of fresh water, sediment, and nutrients into the bay, these factors potentially influence the formation and structure of resident and storm-induced bacterioplankton communities in this ecosystem. It is the largest sheltered body of water in the Hawaiian Islands, with a surface area of 42 km^2^ and an average depth of 9 m [18]. Numerous streams drain into the bay; the largest source of freshwater input is Kaneohe stream in the southern section [19]. While freshwater plumes have been observed to extend to 0.3 km offshore and decrease the salinity from 35.0‰ to 19.3‰ in southern Kaneohe Bay during heavy rainfall events [20], in general this system is characterized by seawater of marine salinity (ca. 35‰) and a very minor, abrupt estuary component. Additionally, there is significant anthropogenic influence on the bay from the densely populated towns of Kaneohe and Kailua that surround it. The bay represents a highly complex pelagic landscape with steep environmental gradients [20].

In this study, high-resolution spatial and temporal sampling and terminal restriction fragment length polymorphism (T-RFLP) analysis combined with small subunit ribosomal RNA (SSU rRNA) gene cloning and sequencing was used to describe the structure of bacterioplankton communities throughout this complex subtropical embayment, and to characterize their response to a natural perturbation (i.e. major storm event). This embayment has been the focus of previous studies (e.g. [16], [17], [19], [20], [21]), and hence represents a good model system to study natural disturbance events.

## Methods

### Sampling

Sampling seawater is not a regulated activity in coastal zones of the State of Hawaii, therefore no specific permits were required for the described field studies. The water sampling described here did not involve endangered or protected species.

**Figure 1:**
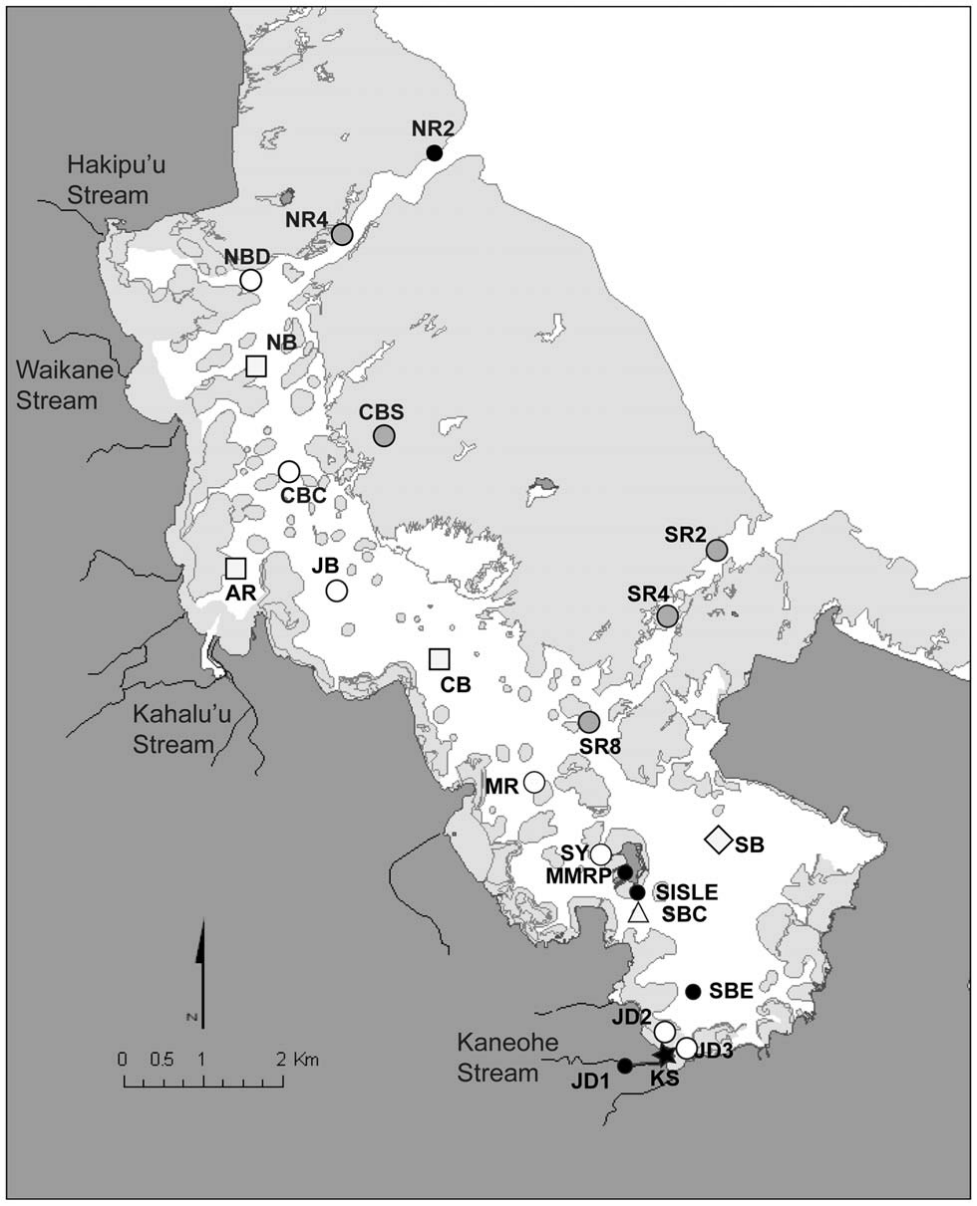
Sampling stations in Kaneohe Bay, Hawaii, included in this study. White symbols indicate stations that clustered into a coastal group, grey symbols indicate stations that clustered into an offshore group, and the black star indicates a single freshwater station, based on K-means clustering analysis. Black circles indicate stations that did not have enough data to include in the K-means cluster analysis. Symbol shapes indicate the type of difference observed between storm and non-storm conditions: circle or star, neither the microbial community structure nor environmental conditions differed significantly; square, both microbial community structure and environmental conditions differed significantly; triangle, only environmental conditions differed; diamond, only microbial community composition differed. Light gray areas indicate shallow patch, fringing and barrier reefs.

Routine sampling was performed every 3 weeks between February and September 2006 at 23 stations throughout Kaneohe Bay, including one station at the mouth of Kaneohe stream (KS), 2 stations in close proximity to the stream mouth (JD2 and JD3), and 1 station upstream (JD1) (Fig. 1, Table S1). At each station, 1.7 L of seawater was collected from a depth of 1 m via a Teflon-lined Niskin bottle (General Oceanics Inc., Miami, FL), and used for DNA-based analyses of microbial community structure, measurement of chlorophyll *a* concentration, flow cytometric enumeration of picoplanktonic cells, and quantification of macronutrients [soluble reactive phosphorus (SRP), nitrate + nitrite (N+N), ammonia (NH_4_^+^), nitrite (NO_2_^−^) and silicic acid (H_2_SiO_4_)]. A multiparameter water quality monitoring sonde (YSI 6600; YSI Incorporated, Yellow Springs, OH) was used to obtain in situ profiles of temperature, salinity, and pH. Precipitation data were measured at the Luluku (Kaneohe stream), Hakipu‘u, and Waikane rain gauges and were obtained from the National Oceanic and Atmospheric Administration (NOAA) database (http://www.prh.noaa.gov/hnl/pages/hydrology.php). Air temperature, wind speed and direction, and solar radiation data were obtained from the Hawaii Institute of Marine Biology, tide data was obtained from the National Oceanic and Atmospheric Association (http://www.tidesonline.nos.noaa.gov/), and stream discharge data was obtained from the U. S. Geological Survey.

In addition to routine sampling, storm sampling was conducted on days 1, 4, 7, 18, and 21 post-storm (March 3, 6, 9, 20, and 23, 2006) at thirteen stations after a major storm event occurred on March 2, 2006 (Fig. 1). This storm event was preceded by routine sampling of all 23 stations on February 26, 2006, and followed by routine sampling on April 9, 2006. Additionally, routine sampling of all 23 stations also occurred ten days post-storm on March 12, 2010, resulting in all stations being sampled at least once immediately after the storm event.

### Genomic DNA extraction

Water for microbial DNA analysis was placed on ice and processed on shore within two hours. Approximately 1 L of each sample was filtered through a 25-mm diameter, 1.6-µm pore-sized microfiber pre-filter (GF/A, Whatman International Ltd., Kent, UK) followed by collection of microbial biomass on a 13-mm diameter, 0.2-µm pore-sized polyethersulfone membrane (Supor 200, Pall Gelman Inc., Ann Arbor, MI). Membrane filters were submerged in DNA lysis buffer (20 mmol L^−1^ Tris-HCl pH 8.0, 2 mmol L^−1^ EDTA pH 8.0, 1.2% v/v Triton X100), and stored at –80°C until further processing [22]. Genomic DNA was extracted using a DNeasy 96 Tissue kit (Qiagen Inc., Valencia, CA) following the manufacturer’s recommended protocol for bacteria.

### T-RFLP analysis of SSU rRNA genes

For terminal restriction fragment length polymorphism (T-RFLP) analysis [23], the general bacterial primers 27F-B-FAM (5’-FAM-AGRGTTYGATYMTGGCTCAG-3’) and 519R (5’-GWATTACCGCGGCKGCTG-3’) [24], were used for the amplification of small subunit ribosomal RNA (SSU rRNA) genes. PCR was performed as follows: 0.625 U of PicoMaxx high fidelity DNA polymerase (Stratagene, La Jolla, CA), PicoMaxx 1X reaction buffer (Stratagene), 200 nmol L^−1^ of each primer, 0.2 mmol L^−1^ of each deoxynucleoside triphosphate (dNTPs), and 10 ng of mixed environmental genomic DNA were combined in a final reaction volume of 50 µL. After an initial denaturation step at 95°C for 5 min, the reaction conditions were: 24 cycles of 95°C denaturation for 30 s, 55°C annealing for 1 min, and 72°C extension for 2 min, concluding with an extension at 72°C for 20 min.

The fluorescently labeled amplicons were purified using the QIAquick PCR purification kit (Qiagen Inc.) following the manufacturer’s instructions, except that the final elution step was repeated to increase yield. Approximately 100 ng of each purified amplicon was subsequently digested in a 10-µL reaction containing 5 units of HaeIII restriction endonuclease (Promega, Madison, WI) at 37°C for 7 hours. After purification via gel filtration chromatography with Sephadex G-50 (Amersham Biosciences, Sweden), the restricted samples were adjusted to a final concentration of 30 ng µL^−1^ and separated via capillary electrophoresis on an automated ABI 3100 Genetic Analyzer (Applied Biosystems, Foster City, CA). GeneMapper software (Applied Biosystems) was used to estimate the size and relative abundance of the resulting terminal restriction fragments (T-RFs), which were defined as fragments between 34 and 600 base pairs (bp) in length. Fragment lengths were rounded to the nearest integer value, aligned, and manually corrected for likely errors in peak determination due to such factors as instrument drift, etc. The threshold below which peaks were excluded was determined via the variable percentage threshold method as described in Osborne et al. [25].

### SSU rRNA gene clone libraries

SSU rRNA gene clone libraries were constructed from samples taken from Station NB on March 12, 2006 (10 days post-storm initiation), and June 28, 2006 (non-storm conditions). SSU rRNA genes were initially PCR-amplified via the following reaction: 0.25 units of PicoMaxx high fidelity polymerase (Stratagene), 1X PicoMaxx reaction buffer (Stratagene), 200 nmol L^−1^ of each of the primers 27F-B and 1492R (5'-GGTTACCTTGTTACGACTT-3’) [24], 0.2 mmol L^−1^ of each dNTP, and 4 ng of mixed environmental genomic DNA were combined in a final reaction of volume of 20 µL. PCR cycling conditions consisted of a denaturation step at 95°C for 5 minutes followed by 26 cycles of 95°C denaturation for 30 sec, 55°C annealing for 1 min, 72°C extension for 2 min, and a final extension step at 72°C for 20 min. Additionally, a 3-cycle reconditioning PCR was performed to help eliminate heteroduplexes [26]. Reconditioning PCR cycling parameters consisted of an initial denaturation step (95°C for 5 min) followed by 2 cycles of 95°C for 30 sec, 55°C for 1 min, 72°C for 2 min, and a final extension at 72°C for 20 min. PCR amplicons were purified using the QIAquick PCR purification kit (Qiagen) and subsequently cloned using the pGEM-T Easy system (Promega) following the manufacturer’s instructions. Plasmids were sequenced bi-directionally using an ABI 3730XL capillary-based DNA sequencer (Applied Biosystems) and M13 primers. The resulting SSU rRNA gene clone sequences were trimmed free of vector sequence and assembled using Sequencher (Gene Codes, Ann Arbor, MI), and then checked for chimera formation using the CHECK_CHIMERA software from the Ribosomal Database Project [27], as well as nearest neighbor comparisons in phylogenies constructed separately from 5’ and 3’ regions of clone sequence. Curated clone sequences were aligned with the SILVA 92 ARB database using the ARB software package [28], which was modified to include environmental gene clones of high similarity to the clone sequences obtained in this study that were published after the release of this database. Identities were determined by adding sequences generated in this study to manually curated guide phylogenies of marine bacterioplankton maintained in ARB. Phylogenetic analyses were constructed using the RAxML maximum likelihood method [29] from nearly full-length gene sequences within ARB, employing sequence alignments generated with the ‘All-Species Living Tree’ project SSU rRNA gene database [30], modified to include previously published environmental gene clones of high similarity to the clone sequences obtained in this study. Bootstrap analyses were determined by RAxML [31] via the CIPRES Portal V 1.15 available online [32].

The probe match tool in ARB was used to predict HaeIII cut sites and subsequent predicted T-RF size of clone sequences. Using the T-RFLP protocol described above for environmental genomic DNA, T-RF lengths were experimentally determined for clones representing the majority of phylogenetic lineages recovered in each library. Thus, empirically determined rather than predicted T-RF sizes were used to match bacterial lineages with T-RFs present in the community T-RFLP profiles.

All sequences generated in this study have been deposited in GenBank under accession numbers KC425475-KC425609 and KC430933.

### Direct cell counts

Flow cytometry was used to enumerate cyanobacteria (*Prochlorococcus* and *Synechococcus* spp.), picoeukaryotic phytoplankton, and non-pigmented prokaryotes. At each sampling station, 1 mL of raw sample was fixed in a final concentration of 1% (v:v) paraformaldehyde and stored at –80°C until analysis. Enumeration was performed on an EPICS ALTRA flow cytometer (Beckman Coulter Inc., Brea, CA), and analysis of the resulting data followed the method of Monger and Landry [33].

### Nutrient and pigment analysis

Dissolved inorganic nutrient concentrations (NH_4_^+^, N+N, NO_2_^−^, SRP, and H_2_SiO_4_) were determined with a continuous segmented flow system consisting of a Technicon AutoAnalyzer II (SEAL Analytical Ltd., Milwaukee, WI) and an Alpkem RFA 300 Rapid Flow Analyzer (Alpkem Corp., Clackamas, OR). Briefly, SRP was measured using a modified molybdenum blue method [34], N+N, NO_2_^−^, and H_2_SiO_4_ analyses were based on Armstrong et al. [35], and NH_4_^+^ was measured using the indophenol blue method [36]. Chlorophyll *a* concentrations were determined by filtering 140 ml of sample water onto 25 mm diameter, 0.7-µm pore-sized glass microfiber filters (GF/F, Whatman). Filters were stored in aluminum foil at –80°C until extraction in 100% acetone and measurement of fluorescence using a Turner 10-AU fluorometer (Turner Designs, Sunnyvale, CA) according to standard techniques [37].

### Statistical analyses of microbial community structure

Statistical analyses were performed using the software packages PRIMER 6 (PRIMER Ltd., Plymouth, UK; [38]) and ‘R’ (http://www.r-project.org/). Non-metric multidimensional scaling (NMDS) was used to visualize patterns in microbial community structure over space and time in Kaneohe Bay. NMDS iteratively seeks the best position of n entities on k dimensions that minimize stress of the final configuration, and has been effectively used to explore and graphically represent relationships in microbial communities (e.g. [4], [39]). Analysis of similarity (ANOSIM) was used to examine the differences of microbial assemblages between stations, and storm (defined as March 3-12, 2006, sampling events) and non-storm samples at each station. Sorenson (Bray-Curtis) distance was used on Spearman rank-transformed T-RFLP data. ANOSIM computes a test statistic (R), where R = 1 if all replicates within a factor (storm and non-storm) are more similar to each other than any replicates from different factors. R is approximately zero if the null hypothesis is true that similarities between and within factors are the same. A Monte Carlo simulation where the Bray Curtis matrix is randomly rearranged allows comparison between simulated and observed R-values and also determines the significance level at which the null hypothesis can be rejected. In conjunction with NMDS and ANOSIM, hierarchical cluster analyses with average-group linking, based on R-values from the pair wise comparison between sites was used for delineating groups of sites with distinct community structure (PRIMER 6). Permutational analysis of variance was run to identify differences in microbial community composition in samples related to storm or non-storm, as well as differences related to stations, and the combined effect. A K-means clustering analysis using the Hartigan–Wong algorithm [40] was also performed to identify groups of stations with similar patterns of microbial community composition over time using an R software package. This analysis was restricted to a subset of the sampling sites due to missing data, which are indicated in Figure 1.

A BIO-ENV procedure [41] was used to identify environmental variables or sets of variables that best match the T-RFLP patterns. This procedure assembles all combinations of environmental variables into Euclidean distance matrices. In a second step, it determines rank correlation between the Bray-Curtis T-RFLP data and the Euclidean environmental distance matrix using the Spearman coefficient (r). Correlations are ranked by r-values and the best matching variable combinations are chosen. RELATE test was used to determine *p*-values, still such significance tests based on *a priori* selection of variables are biased.

Stations that were found to have distinct storm and non-storm bacterioplankton community structure via ANOSIM analysis were further examined using SIMPER analysis. SIMPER analysis identified the T-RFLP peaks that contributed the most to the dissimilarity between storm and non-storm communities. The SIMPER analysis decomposed the average Bray Curtis dissimilarities between all pairs of samples into percentage contributions from each T-RFLP peak. Thus, SIMPER analysis identified the T-RFLP peaks that contributed the most to the dissimilarity between storm and non-storm communities.

## Results

### General characteristics of Kaneohe Bay during non-storm conditions

Outside of two stations directly influenced by the freshwater of Kaneohe stream (JD1 and KS; Fig. 1, Table S1), water temperature and salinity varied little throughout the bay on any individual non-storm or post-storm sampling day (Table 1). Salinity remained uniform at 34 to 35 throughout the bay and, although median surface seawater temperature fluctuated between 22–30°C during the course of this study, it generally fluctuated no more than 2–3°C on any individual sampling day (Table 1 and data not shown). One notable exception was station AR, where salinity was sporadically depressed to 15 to 30 presumably due to freshwater input from either Kahalu’u stream nearby, or Hakipu‘u and Waikane streams slightly to the north (data not shown).

**Table 1:** Chemical and biological characteristics at sampling stations within and near Kaneohe Bay, Hawaii, over the course of this study.

	Salinity	SRP	NH_4_^+^	N+N	NO_2_^−^	H_2_SiO_4_	Chl. *a* ^a^	Synecho.^a^	Hetero.^a^	Picoeuk.^a^
Station	(‰)	(µmol L^−1^)	(µmol L^−1^)	(µmol L^−1^)	(µmol L^−1^)	(µmol L^−1^)	(µg L^−1^)	(×10^5^ cells mL^−1^)	(×10^6^ cells mL^−1^)	(×10^4^ cells mL^−1^)
**AR**	32.8 (15.7)^b^	0.13 (0.88)	0.25 (2.27)	0.6 (12.6)	0.13 (0.46)	27.0 (147.6)	2.2 (8.4)	1.6 (3.4)	1.5 (3.1)	1.8 (16.7)
**CB**	34.3 (28.1)	0.08 (0.34)	0.08 (0.24)	0.2 (3.2)	0.09 (0.30)	5.4 (14.5)	1.9 (2.6)	1.3 (3.5)	1.2 (2.2)	1.2 (3.4)
CBC	34.2	0.06	0.07	0.2	0.11	7.2	2.2	1.5	1.0	1.3
**CBS**	35.0 (28.1)	0.08 (0.36)	0.09 (0.29)	0.2 (1.7)	0.10 (0.17)	1.5 (48.7)	1.5 (2.5)	1.6 (6.3)	0.7 (0.4)	0.4 (2.1)
JB	34.4	0.08	0.06	0.2	0.08	6.2	2.1	1.3	1.2	1.7
JD1	0.4	0.41	1.37	16.0	0.27	369.8	3.0	0.0	0.8	1.2
JD2	33.9	0.19	0.32	0.6	0.15	16.2	5.7	2.2	2.1	2.8
JD3	34.4	0.16	0.57	0.3	0.14	11.5	3.3	1.8	2.1	2.1
**KS**	23.0 (1.5)	0.54 (2.40)	2.42 (11.24)	20.7 (50.2)	0.29 (0.53)	124.1 (343.6)	2.2 (14.1)	0.2 (1.7)	1.1 (3.0)	1.3 (6.4)
MMRP	34.8	0.10	0.31	0.7	0.14	9.8	2.3	1.0	1.1	1.6
MR	34.7	0.07	0.08	0.2	0.09	7.4	2.1	1.6	1.3	1.9
**NB**	34.0 (21.0)	0.10 (0.98)	0.15 (12.69)	0.4 (12.4)	0.15 (0.66)	10.7 (68.4)	2.1 (18.5)	0.7 (2.6)	1.0 (3.6)	1.1 (3.2)
NBD	34.0	0.06	0.07	0.2	0.10	8.0	2.1	0.9	1.2	1.1
**NR2**	35.0 (34.0)	0.10 (0.26)	0.17 (0.41)	0.4 (2.3)	0.11 (0.28)	3.0 (13.5)	1.7 (2.1)	0.3 (1.6)	0.7 (1.2)	0.6 (1.8)
NR4	34.9	0.06	0.04	0.3	0.10	3.3	1.9	0.7	0.9	0.7
**SB**	35.0 (33.5)	0.10 (0.24)	0.07 (0.17)	0.2 (1.2)	0.08 (0.20)	5.7 (10.0)	2.4 (1.7)	2.1 (3.9)	1.4 (3.2)	2.5 (4.4)
**SBC**	35.0 (30.0)	0.10 (0.76)	0.14 (1.88)	0.4 (7.8)	0.10 (0.58)	8.6 (38.7)	2.4 (7.5)	1.6 (2.8)	1.6 (2.8)	2.1 (5.9)
**SBE**	35.0 (31.0)	0.13 (0.47)	0.14 (0.50)	0.2 (2.4)	0.10 (0.24)	7.9 (15.0)	2.8 (6.9)	2.2 (4.5)	1.9 (1.0)	3.2 (1.0)
**SISLE**	35.0 (22.0)	0.10 (1.34)	0.08 (2.12)	0.3 (8.1)	0.09 (0.37)	6.3 (37.1)	2.3 (0.5)	1.6 (4.6)	1.4 (2.3)	2.2 (4.5)
**SR2**	35.0 (34.1)	0.08 (0.15)	0.05 (0.18)	0.2 (0.5)	0.10 (0.12)	1.2 (3.6)	1.5 (1.8)	3.0 (6.6)	0.7 (0.5)	0.6 (1.1)
SR4	35.0	0.07	0.06	0.2	0.09	1.3	1.4	0.3	0.8	0.6
**SR8**	35.0 (34.0)	0.08 (0.53)	0.06 (0.70)	0.3 (2.1)	0.09 (0.32)	4.6 (11.3)	1.8 (2.7)	1.1 (3.1)	1.0 (2.4)	1.4 (2.8)
SY	34.9	0.08	0.10	0.2	0.09	8.3	2.3	1.2	1.5	1.9

a. Chl. *a* - Chlorophyll *a*; Synecho. - *Synechococcus*; Hetero. - non-pigmented prokaryotes; Picoeuk. - picoeukaryotic phytoplanktonb. Median values are listed for all stations. Stations sampled at high frequency throughout the storm and post-storm period (March 3–12, 2006) are listed in bold. Values in parentheses are the greatest deviance from median values during the storm and post-storm perio

Inorganic nutrient concentrations varied widely across Kaneohe Bay during non-storm conditions: SRP concentrations generally ranged from 0.03–0.30 µmol L^−1^, N+N ranged from 0.06–2.0 µmol L^−1^, NO_2_^−^ ranged from 0.06–0.30 µmol L^−1^, NH_4_^+^ ranged from 0.02–0.70 µmol L^−1^, and H_2_SiO_4_ ranged from 0.7–50 µmol L^−1^ (Table 1, Fig. S1). Inorganic nutrients generally followed a similar trend to salinity in that stations impacted by freshwater input exhibited elevated levels of inorganic nutrients, while outer bay stations NR2, SR2, and SR4 were uniformly on the low end of the range (Fig. S1 and data not shown). Additionally, H_2_SiO_4_ and NH_4_^+^ tended to vary widely for stations in close proximity to land (Table 1).

A fairly consistent distribution of chlorophyll *a* concentrations was observed during non-storm conditions. At the majority of stations across the bay, chlorophyll *a* ranged from 1.0–3.0 µg L^−1^. At outer bay stations NR2, SR2, SR4, and barrier reef station CBS, chlorophyll *a* concentrations rarely exceeded 2.0 µg L^−1^ (Table 1). Chlorophyll *a* concentrations were generally elevated (>3.0 µg L^−1^) at stations JD1, JD2, JD3, and KS in close proximity to Kaneohe stream, and fluctuated broadly for stations AR (1.2–8.4 µg L^−1^), CBC (1.5–17.3 µg L^−1^), SBC (1.6–7.5 µg L^−1^), and SBE (1.9–6.9 µg L^−1^) (Table 1, Fig. S1).

Cellular abundances of *Synechococcus* spp., non-pigmented, putatively heterotrophic prokaryotes, and picoeukaryotic phytoplankton were spatially and temporally dynamic during non-storm conditions. *Synechococcus* spp. cellular abundances ranged from 1.1×10^3^ to 4.6×10^5^ cells mL^−1^, non-pigmented prokaryotes ranged from 0.2–3.9×10^6^ cells mL^−1^, and picoeukaryotic phytoplankton ranged from 0.2–16.7×10^4^ cells mL^−1^. In general, cellular abundances were higher along the coastline and decreased offshore (Table 1, Fig. S1), and were also elevated in the southern portion of the bay near the mouth of Kaneohe stream relative to the rest of the bay.

### General differences between storm and post-storm conditions

**Figure 2:**
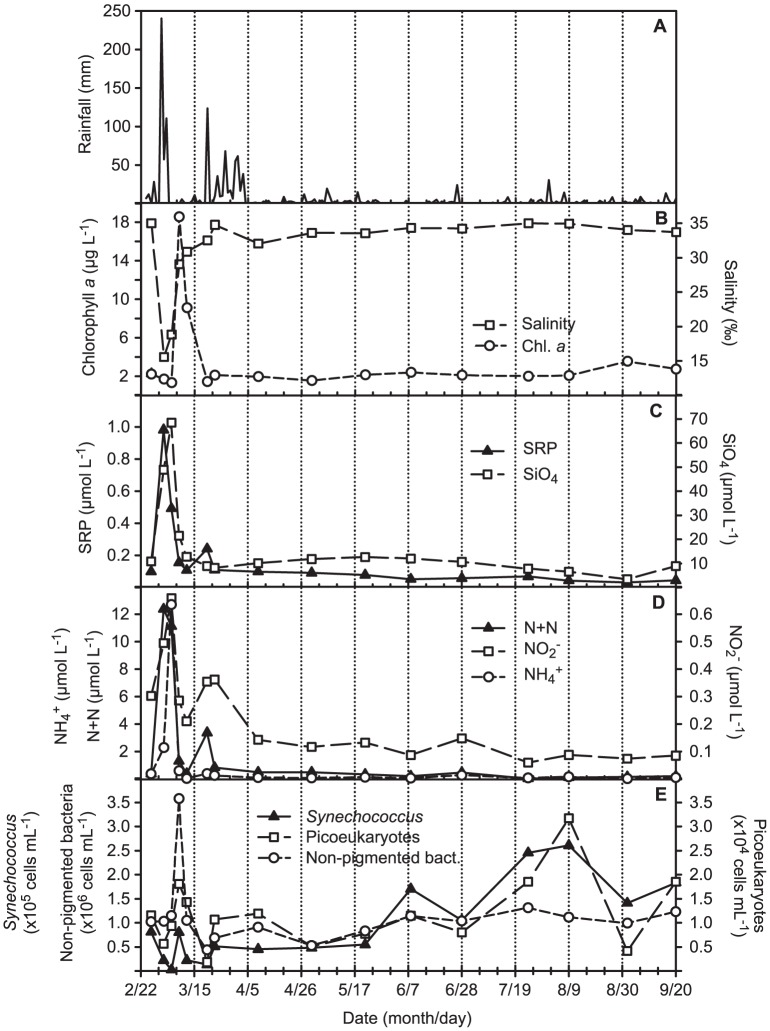
Temporal characteristics of station NB from February to September, 2006. Shown are temporal trends in (A) 24-hour total rainfall measured by the Hakipu’u rain gauge, (B) salinity and chlorophyll *a* concentrations, (C and D) inorganic nutrient concentrations, and (E) cellular abundances.

Heavy rains fell over the Kaneohe Bay watershed on March 2, 2006. Rain gauges at Hakipu‘u and Waikane streams in the northern bay received a total of 240.0 mm and 177.8 mm of precipitation in 24 hours, respectively (Fig. 2 and data not shown). The following day, the Luluku rain gauge at Kaneohe stream in the southern bay recorded a 24-hour precipitation total of 99.8 mm (data not shown). This large volume of precipitation, and consequent runoff into Kaneohe Bay, resulted in large increases in inorganic nutrient concentrations, chlorophyll *a*, and non-pigmented prokaryotic cells in certain regions of the bay (Table 1, Figs. S1 and 2). A lag period of 3 days was observed between the injection of inorganic nutrients and a biological response in the form of increased chlorophyll *a* concentration and non-pigmented prokaryotic cell abundance: SRP (1.0 µmol L^−1^), N+N (12.4 µmol L^−1^), H_2_SiO_4_ (68.4 µmol L^−1^), and NH_4_^+^ (12.7 µmol L^−1^) reached peak concentrations 1–4 days post-storm (March 3–6, 2006; e.g. Fig. 2 from station NB), while chlorophyll *a* concentrations (18.5 µg L^−1^) and non-pigmented prokaryotic cell abundance (3.6×10^6^ cells mL^−1^) peaked seven days post-storm (March 9, 2006). *Synechococcus* spp. (8.0×10^4^ cells mL^−1^) and picoeukaryotic phytoplankton (1.8×10^4^ cells mL^−1^) abundances also increased after an 8-day lag following the storm, though the response was muted in comparison to the non-pigmented prokaryotes. Despite intermittently high rainfall in the Kaneohe Bay watershed throughout March and early April 2006 (e.g. Fig. 2 from the Hakipu’u rain gauge), inorganic nutrient concentrations returned to or near non-storm levels by 10 days post-storm (March 12, 2006). By 18 days post-storm (March 20, 2006), chlorophyll *a* concentrations and non-pigmented prokaryotic cell abundance also returned to non-storm levels (Fig. 2). The environmental data were transformed into a Euclidean distance matrix and, after standardization, analyzed via ANOSIM, which revealed significant differences in storm (hereafter defined as sampling dates from March 3–12, 2006) versus non-storm environmental conditions at stations AR, CB, NB and SBC (1 way ANOSIM, p<0.05 in Table 2).

**Table 2:** Results of ANOSIM testing the null hypothesis that there were no differences in environmental conditions (R_env_, p_env_)^ab^ or community structure (R_com_, p_com_)^ac^ during storm and non-storm conditions in Kaneohe Bay.

Station	R_env_	p_env_	R_com_	p_com_	Ratio
**AR^d^**	0.464	**0.025**	0.426	**0.007**	0.92
**CB**	0.492	**0.011**	0.508	**0.011**	1.03
CBC	0.074	0.400	0.019	0.700	0.26^e^
**CBS**	0.217	0.091	0.098	0.264	0.45
JB	−0.105	0.600	0.006	0.500	0.06^e^
JD1^f^	−	−	−	−	−
JD2	−0.061	0.375	0.32	0.250	5.25
JD3	−0.102	0.500	0.646	0.125	6.33
**KS**	0.017	0.427	−0.115	0.815	6.76^e^
MMRP	−0.247	0.800	0.341	0.089	1.38
MR	−0.107	0.556	0.375	0.222	3.50
**NB**	0.444	**0.022**	0.882	**0.002**	1.99
NBD	0.241	0.300	0.883	0.100	3.66
**NR2**	0.318	0.057	0.347	0.068	1.09
NR4	0.136	0.400	−0.006	0.500	0.04^e^
**SB**	0.031	0.318	0.568	**0.024**	18.32
**SBC**	0.366	**0.018**	0.114	0.210	0.31
**SBE**	0.222	0.125	−0.02	0.508	0.09
**SISLE**	0.234	0.125	0.012	0.442	0.05
**SR2**	0.115	0.225	0.052	0.358	0.45^e^
SR4	−0.241	0.778	−0.054	0.444	0.22
**SR8**	0.155	0.227	−0.043	0.559	0.28^e^
SY	1	0.143	0	0.571	0.00

a. R - test statistic; p - significance value (in bold are significant at <0.05) b. Based on a Euclidean distance matrix of environmental conditions including temperature, salinity, NH_4_^+^, NO_2_^−^, N+N, and SRP. c. Based on a Bray-Curtis distance matrix calculated from T-RFLP community composition data. d. Stations listed in bold were sampled intensively throughout the storm and post-storm period, and thus have four sample dates included in the comparison. All other samples have one sampling event included in the comparison (see Materials and Methods) e. Measure should be taken with caution because both R-values are near zero. f. Station JD1 was not sampled from March 3–12, 2006, and thus has no data obtained from storm conditions

Two-way crossed analysis of similarity ANOSIM (stations and storm vs. non-storm conditions as factors) was performed to test for differences in environmental variables between the stations. Global ANOSIM revealed differences between the stations (R = 0.055 and p<0.01; excluding storm samples, R = 0.088 and p<0.001) and also between the overall storm and non-storm samples (R = 0.237, p<0.01) based on a Euclidean matrix derived using mostly chemical environmental parameters (temperature, salinity, SRP, N+N, H_2_SiO_4_, and NH_4_^+^). Pair-wise comparisons revealed that stations JD1, KS, SR2, and SR4 were the stations most responsible for observed heterogeneity throughout the bay.

#### General microbial community dynamics

Microbial community composition was assessed by T-RFLP analysis of bacterial SSU rRNA genes in 244 surface water samples collected over the 8-month sampling period. A total of 288 distinct terminal restriction fragments (T-RFs) were detected, with an average of 52.2 T-RFs per sample. Only one of the 288 distinct T-RFs detected in this study was recovered in all 244 samples (34 base pairs in length) though several other T-RFs were found in at least 80% of the samples, including fragments of 113, 135, 186, 187, 221, 226, 289, and 327 bp.

**Figure 3:**
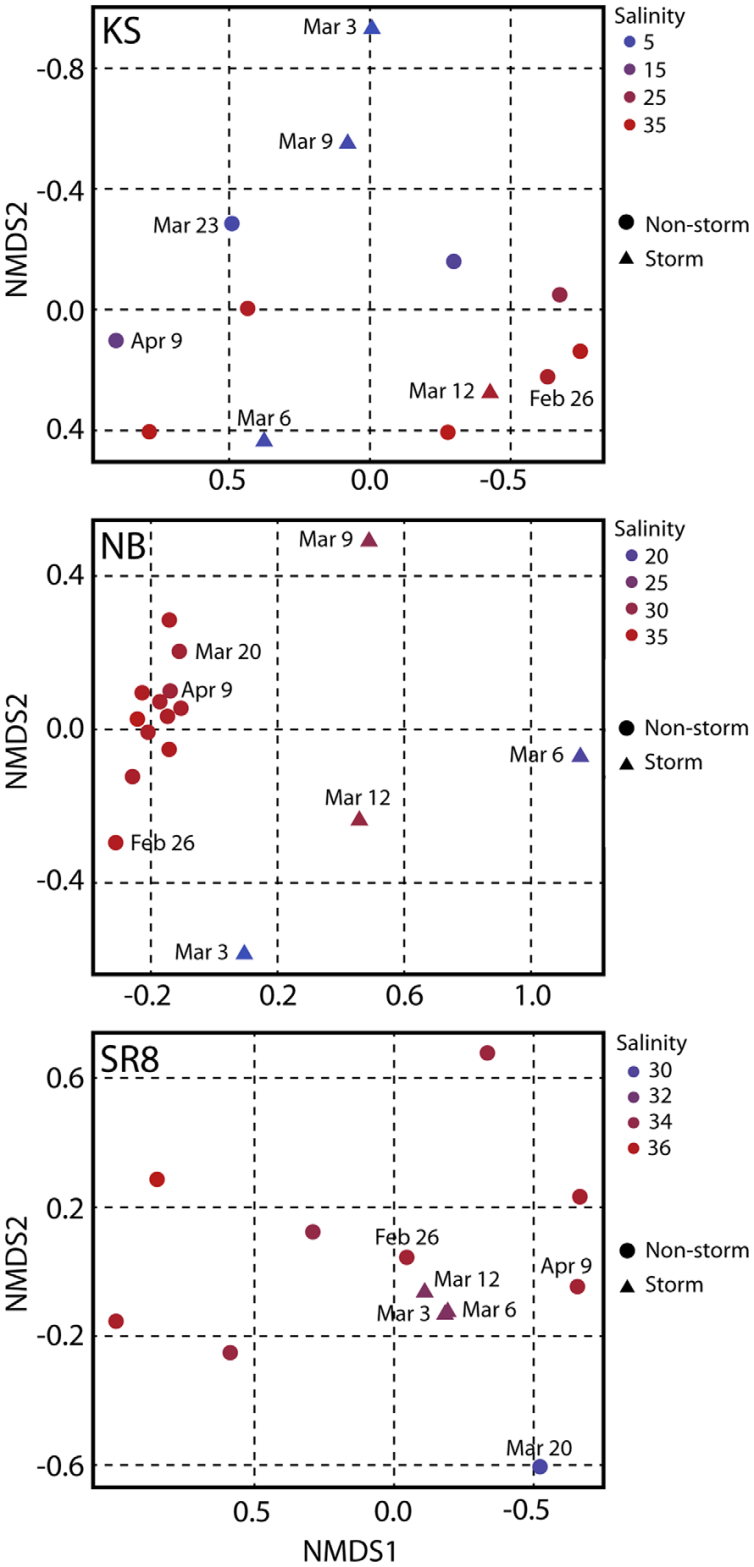
Non-metric multidimensional scaling (NMDS) analyses of representative stations from the three groups identified using K-means cluster analysis. Bacterial community structure from water sampled at stations representing freshwater (KS), coastal (NB), and offshore (SR8) sample site groups are shown.

The dynamics of the microbial community structure at sites within the bay were further assessed using a K-means clustering analysis. This analysis revealed that sites fell into 3 groups based on the dynamics of their microbial community structure over the time period of the study. These groups were an ‘offshore’ cluster (sites CBS, NR4, SR2, SR4, and SR8), a ‘coastal’ cluster (sites AR, CB, CBC, JB, JD2, JD3, MR, NB, NBD, SB, SBC, and SY), and a freshwater influenced site (KS) (Figs. 1 and 3). Sites not listed here were not included in the K-means analysis due to missing data. Comparable results were found using non-metric multidimensional scaling (NMDS) of the T-RFLP community profile data: NMDS results indicated that for the most part, samples originating from stations JD1 and KS in freshwater Kaneohe Stream clustered separately from all other stations, while offshore/outer bay stations SR2, SR4, NR2, NR4 and CBS clustered together (Fig. S2). These clusters of bacterioplankton communities were corroborated by significant differences in pair-wise ANOSIM between individual stations during non-storm conditions (data not shown).

Using BIO-ENV procedures, the correspondence between microbial community structure and environmental parameters was analyzed. The parameter with the highest correspondence was H_2_SiO_4_ (r = 0445, p<0.001), followed by the combination of N+N and H_2_SiO_4_ (r = 0.432, p<0.001). Other environmental variables that significantly corresponded to the observed microbial community dynamics were salinity (r = 0.281, p<0.001), NH_4_^+^ (r = 0.328, p<0.001) and SRP (r = 0.297 p<0.001). Other abiotic parameters including tidal height (p = 0.14) and temperature (r = 0.108, p = 0.002) showed only low or insignificant correspondence with the surface bacterioplankton community structure. These relationships were maintained when BIO-ENV procedures were performed separately with samples from each of the three k-means clusters (offshore, coastal, and freshwater).

### Effect of storm event on microbial community dynamics

**Figure 4:**
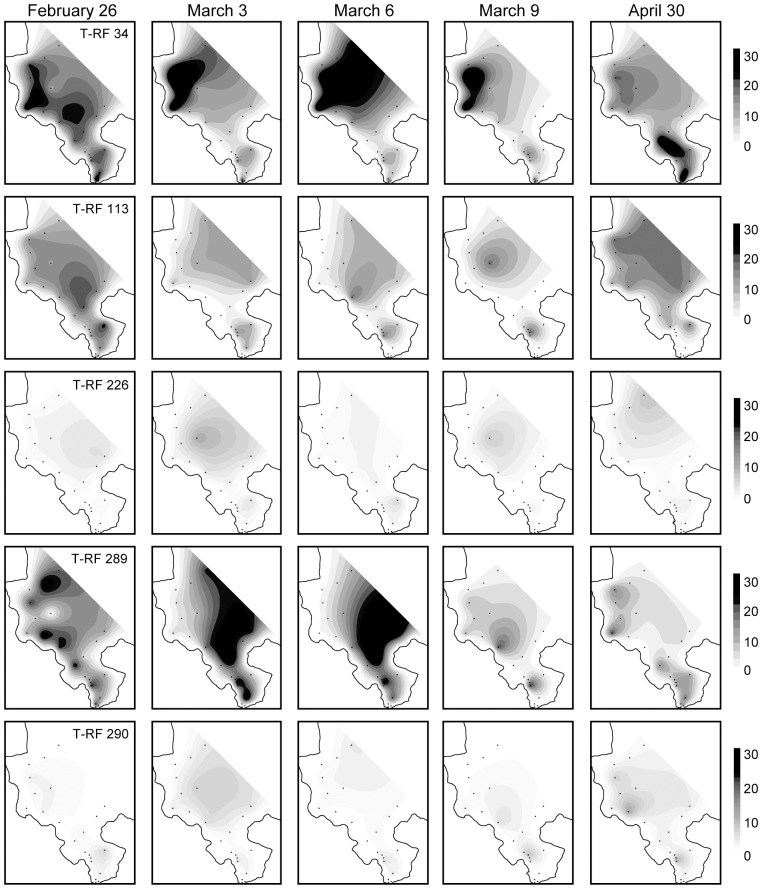
Temporal variation in T-RFs across Kaneohe Bay. Contour plots showing temporal variation in the relative abundance of SSU rRNA gene terminal restriction fragments with the highest contribution to the dissimilarity between storm and non-storm conditions throughout Kaneohe bay immediately prior to (February 26), during (March 3–9), and after (April 30) the March 2006 storm event.

Of the thirteen sites that were sampled intensively immediately following the storm event, four sites were found to have significant differences in their microbial community structure between storm and non-storm conditions (1-way ANOSIM, p<0.05, Figs. 1 and S3, Table 2). All four of these sites fell within the coastal cluster identified through the K-means clustering analysis. Three of these four sites also had significantly different environmental conditions following the storm, based on ANOSIM results reported above (stations AR, CB and NB; Table 2, Fig. 1). Station SB also had a significantly different microbial community following the storm event, but did not demonstrate correspondingly different environmental conditions following the storm (Table 2). In contrast, station SBC possessed different environmental conditions (1-way ANOSIM, p<0.05), but community structure between storm and non-storm samples was not significantly different (Table 2). Station NB showed the greatest difference between storm and non-storm microbial community composition (R-value 0.882) and, at all four stations that had a significantly different microbial community following the storm, the microbial communities showed the most pronounced difference from average conditions on March 6, 2006 (5 days post-storm; Fig. S3). Moreover, as exemplified for stations NB (Fig. 3) and stations AR, CB, and SB (Fig. S3), the bacterial communities returned to compositions highly similar to pre-storm communities by the March 20 or April 9 sampling dates. This recovery is also evident in contour plots of several different T-RFs across the entire bay (Fig. 4).

SSU rRNA gene clone libraries were used to determine the phylogenetic affiliation of microbial assemblages found at Station NB during non-storm conditions (June 28, 2006; clone library prefix ‘NB62806’), as well as immediately after the storm (March 12, 2006; clone library prefix ‘NB31206’). In addition, we sought to match common T-RFs recovered from the extensive T-RFLP analysis to gene clone sequences. A total of 356 SSU rRNA gene clones were sequenced: 182 from NB62806 and 174 from NB31206 (Table S2).

All of the T-RFs that were found in at least 80% of the T-RFLP profiles were putatively identified in one or both clone libraries. These T-RFs corresponded to well-known and ubiquitous lineages of marine bacterioplankton such as SAR11 subclades IA (T-RF 113), IB (T-RF 226), and II+III (T-RF 290), marine *Synechococcus* spp. (T-RFs 135 and 289), the uncultivated SAR86 lineage of *Gammaproteobacteria* (T-RFs 186 and 187), the marine *Actinobacteria* clade (T-RF 327), and the coastal betaproteobacterial clade OM43 (T-RF 221). SSU rRNA gene clones related to the *Gammaproteobacteria*, *Bacteroidetes*, and the *Rhodobacterales* of the *Alphaproteobacteria* were discovered to possess a T-RF of 34 bp, indicating it was polyphyletic (Table 3 and data not shown).

**Table 3:** Summary of 16S rRNA gene clone groups differentially recovered from station NB during storm and non-storm conditions.

	Percent recovery from:		
Phylogenetic affiliation	Storm^a^	Non-storm^b^	T-RF (bp)^c^
*Alphaproteobacteria*, *Rhodobacterales*	42.8	20.2	34
*Betaproteobacteria*, uncultured *Comamonadacae*	12.7	0	197
*Alphaproteobacteria*, SAR11 subgroup IA	5.8	1.6	113
*Cyanobacteria*, *Prasinophyceae* chloroplast	5.8	2.7	383
*Gammaproteobacteria*, *Alteromonas*	2.9	0	34
*Cyanobacteria*, *Bacillariophyta* chloroplast	0	3.3	377
*Actinobacteria*, Marine *Actinobacteria* clade	0.6	2.7	327
*Alphaproteobacteria*, SAR116	1.2	4.9	134, 225
*Gammaproteobacteria*, OM60	1.7	4.9	34
*Betaproteobacteria*, OM43	3.5	6.0	221
*Bacteriodetes*, unclassified *Flavobacteriales*	1.2	10.9	34
*Cyanobacteria*, *Synechococcus* spp.	1.2	24.0	135, 289

a. Storm sampled March 12, 2006. b. Non-storm sampled June 28, 2006. c. Actual terminal restriction fragment length, as determined from representative clones. Two values indicate clone groups containing two prevalent T-RFs

Based on a SIMPER analysis, T-RFs of 34, 113, 226, 289, and 290 bp were found to contribute most highly to the dissimilarity between storm and non-storm communities at stations NB, AR, CB and SB (Table 4). These T-RFs exhibited dynamic, heterogeneous responses to the storm event that frequently differed between stations (Figs. 1 and 4). For example, at nearby stations NB and AR, T-RF 34 increased markedly during the storm event, and subsequently returned to non-storm values almost immediately after the storm. This coincided with a rapid increase in the cellular abundance of heterotrophic bacteria (Table 1, Fig. 2). However, at stations CB and SB, this T-RF exhibited a more muted response, and instead exhibited a stronger response from T-RF 113 that was similar to offshore station NR2 (Fig. 4). Most stations exhibited a decrease in the relative abundance of T-RF 289 (marine *Synechococcus* spp.) during storm conditions (e.g. Fig. 4). However, the cellular abundance of *Synechococcus* spp. actually increased during this time period; the T-RFLP results accurately reflect that it’s relative contribution to total cell counts decreased because of the more intense blooming of heterotrophic bacteria (Fig. 2). This is in contrast to summer increases in the cellular abundance of *Synechococcus* spp. across the bay that are not accompanied by an increase in the cellular abundance of heterotrophic bacteria (e.g. Fig. 2 for station NB), which result in a higher relative contribution of T-RF 289 to the total bacterioplankton community (data not shown).

**Table 4:** Terminal restriction fragments with the highest contributions to the dissimilarity between storm and non-storm conditions at stations AR, CB, NB, and SB, based on SIMPER.

		Response to storm at station:			
T-RF	Phylogenetic affiliation	AR	CB	NB	SB
34	*Alphaproteobacteria*, *Rhodobacterales*; *Gammaproteobacteria*; *Bacteroidetes*	+(21.8)^a^	−(8.5)	+(25.4)	−(6.0)
113	*Alphaproteobacteria*, SAR11 subgroup IA	+(8.9)	+(17.5)	−(7.0)	+(12.4)
226	*Alphaproteobacteria*, SAR11 subgroup IB	−(1.0)	+(2.4)	−(1.3)	+(3.1)
289	*Cyanobacteria*, marine *Synechococcus*	−(20.5)	−(28.6)	−(12.0)	−(16.6)
290	*Alphaproteobacteria*, SAR11 subgroups II&III	−(2.7)	+(2.7)	−(3.8)	−(3.2)

a. Plus (+) symbol indicates an increase in abundance during storm events, while a minus (−) indicates a decline. The relative contributions to the dissimilarity between storm and non-storm samples are shown.

**Figure 5:**
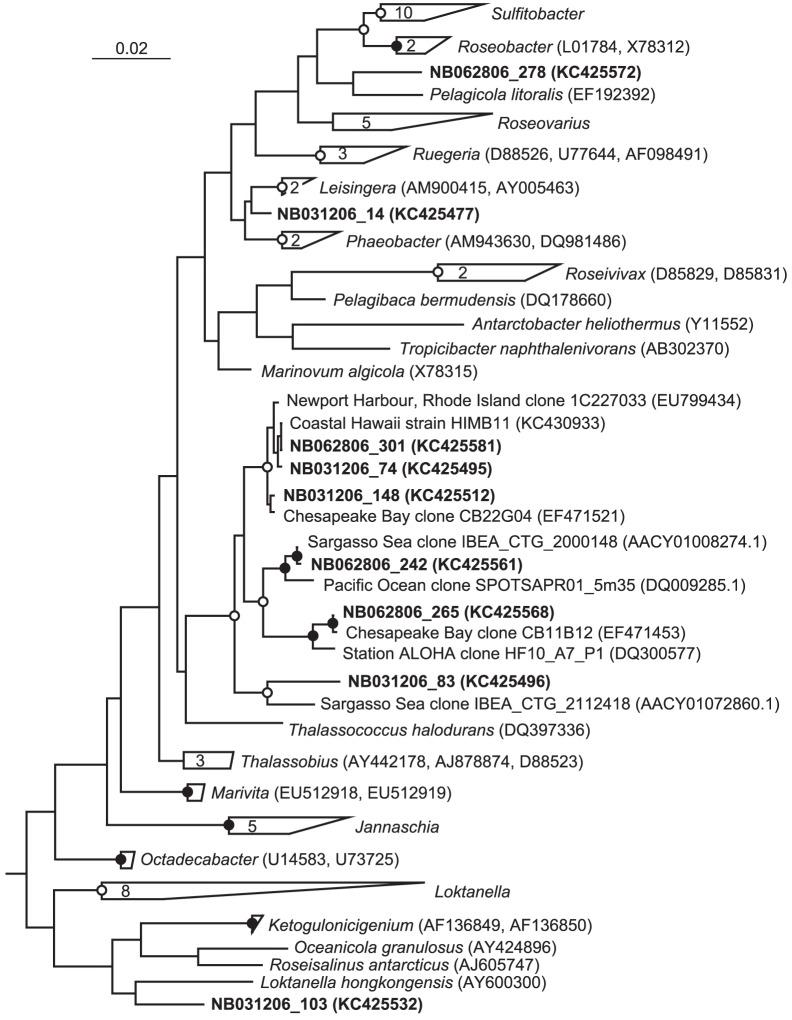
Phylogenetic analysis of *Rhodobacteraceae* recovered from Kaneohe Bay. Phylogenetic relationships between SSU rRNA gene clone sequences obtained from Station NB in Kaneohe Bay (prefixes NB031206 and NB062606) and representatives of the Family *Rhodobacteraceae* of the *Alphaproteobacteria*. The scale bar corresponds to 0.02 substitutions per nucleotide position. Open circles indicate nodes with bootstrap support between 50–80%, while closed circles indicate bootstrap support >80%, from 450 replicates. The bracket indicates the specific lineage primarily responsible for differences between storm and non-storm *Rhodobacteraceae* relative abundances. A variety of *Gammaproteobacteria* were used as outgroups (not shown). Accession numbers for sequences included in the phylogenetic tree but not listed in the figure include: *Sulfitobacter* - AJ550939, Y16425, AY180103, EF202614, AY180102, Y16427, DQ097527, DQ683726, Y17387, Y13155; *Roseovarius* - EU156066, AJ534215, AF098495, DQ120726, Y11551; *Jannaschia* - EF202612, AJ438157, DQ643999, AJ748747, AY906862; *Loktanella* - AY682198, AB246747, AJ582225, DQ344498, EF202613, AY682199, AJ440997, AJ582226.

Several clone groups differed markedly in relative abundance between the storm and non-storm clone libraries constructed from station NB (Table 3, Table S2). In particular, a diverse array of lineages within the Family *Rhodobacteraceae* of the *Alphaproteobacteria* accounted for nearly 43% of the gene clone sequences recovered from the storm sample, as opposed to 20% in the non-storm sample (Table 3, Table S2, Fig. 5). However, one lineage accounted for the majority of clones recovered within the *Rhodobacteraceae* (Fig. 5). This group possessed a T-RF of 34 bp, which is consistent with the T-RFLP results shown above. An uncultivated lineage within the family *Comamonadaceae* of the *Betaproteobacteria* also exhibited a >10% increase in relative abundance in the storm clone library (Table 3 and S2, Fig. S4). While the T-RF corresponding to this group (197 bp) was not found to contribute to the dissimilarity between storm and non-storm communities at station NB as highly as others via SIMPER analysis, it followed the same trend revealed by the clone library analysis, progressing from undetectable prior to the storm event to a maximum of 7.2% of the microbial community during the storm (data not shown). Finally, several lineages within the cyanobacterial genus *Synechococcus* were collectively >20% more abundant in the non-storm clone library (Table 3, Table S2). As previously mentioned, this is consistent with results from flow cytometrically-determined cellular abundance data (Fig. 2) and T-RFLP.

## Discussion

Spatially heterogeneous microbial communities were a regular feature of the subtropical embayment investigated in this study. However, three types of sites - offshore, coastal and freshwater - encapsulated the heterogeneity within the bay. The structure of microbial communities within the three distinct groups appeared likely to be driven by a combination of three prominent characteristics of this environment: highly localized freshwater input, physical forcing due to wind convection, and complex bay topography. Freshwater inlets bring about steep gradients in inorganic nutrient concentrations during non-storm conditions, while the northeasterly trade winds regularly experienced by the Kaneohe Bay watershed consistently confine freshwater to the shoreline [20] and subject offshore stations to mixing with open ocean oligotrophic water. Complex fringe, patch and barrier reefs physically disrupt these predominant environmental forcings, though the reef system may also directly influence the structure of planktonic marine microbial communities within the bay [42].

The major storm event investigated here elicited a rapid cascade of biological responses resulting from a similarly rapid input of inorganic nutrient-rich freshwater runoff, including a major phytoplankton bloom one week after the initiation of the storm. Interestingly, only a subset of stations within the coastal cluster of sampling sites experienced a significant shift in microbial community structure in response to the natural perturbation. In general, differences in environmental conditions were tightly coupled with differences in microbial community structure. Consistent with this observation, inorganic nutrient concentrations were identified as the environmental variables with the greatest correspondence to changes in bacterioplankton community structure immediately after the storm. This was also the case when samples from the ’offshore,’ ‘coastal,’ and ‘freshwater’ clusters were analyzed separately, indicating that the variability described by environmental factors does not only refer to the differences between these three clusters. Thus, the magnitude of cascading biological responses seen at stations NB and AR appears to be due to the increased inorganic nutrient input resulting from the heavy precipitation experienced by the northern portion of the bay relative to the southern portion.

We speculate that disturbances, such as storm events, may play a crucial role in maintaining microbial diversity, as has been suggested for macroorganisms [43]. The combination of T-RFLP and microbial cell abundance data allow us to infer that particular bacterial lineages bloom or invade in a highly localized, site-specific manner, while other lineages decrease or retreat as a result of disturbance. This suggests that habitat diversity and connectivity play crucial roles in maintaining the microbial diversity of this system. Hence, the character of the microbial response may not only be a direct result of the physical and chemical dynamics induced by the disturbance [44], but also might result from characteristics of the localized communities themselves, such as their resistance (i.e. the ability to withstand disturbance) and resilience (the ability to recover after disturbance) [45], [46].

In an attempt to test for the resistance of microbial communities, we used the R-value of the ANOSIM analyses as an estimate for the difference between non-storm and storm community structure (R_com_). R_com_ values were highly variable throughout the bay, with highest values close to the shoreline and decreased with increasing distance from shore. R-values resulting from the environmental dataset (R_env_) represent a proxy of the disturbance strength at each site. By calculating the ratio between R_com_ and R_env_, we attempt to determine the resistance of each community to the disturbance at each site throughout the bay. Ratios close to zero indicate high resistance, whereas values around 1, as observed in most cases, indicate intermediate resistance of the microbial community. Ratios above 1.5 can be interpreted as low resistance, such as was observed at sites strongly influenced by run-off. Alternatively, high ratios can also be the result of high rates of dispersal when environmental conditions stay rather constant (R_env_ close to zero), but migration of organisms due to disturbance induced mixing causes shifts in community structure. This might be the case for station SB, where we observed a significant difference between the microbial community present during storm and non-storm conditions, but the R_env_ was close to zero. High ratios may also be explained by our use of a limited number of environmental variables in the ANOSIM analysis used to determine R_env_, as important parameters could have been missed. Future studies are needed to confirm if this ratio can provide an accurate measure of the resistance of communities.

An increase in heterotrophic bacterial cells generally coincided with the bloom in phytoplankton one week after storm initiation. At station NB, clone library and T-RFLP analysis revealed that this increase partially resulted from an increase in two groups of bacteria: the marine Roseobacter clade of *Alphaproteobacteria*, and a unique lineage of *Betaproteobacteria*. A similar trend in T-RFLP profiles was observed for station AR. The same lineage of marine roseobacters also appeared to be common during non-storm conditions, but at less abundance. Members of the marine Roseobacter clade are common in marine surface waters [47], [48], and are often found in greater abundance in association with phytoplankton blooms (e.g. [49], [50]) and increased nutrients (e.g. [51]). The particular lineage represented by the bulk of marine Roseobacter clade sequences retrieved in this study is closely related to environmental gene clone sequences recovered from a variety of pelagic marine environments, including the North Pacific subtropical gyre near Hawaii [52], the southern California coast [53], and Chesapeake Bay (lineage ‘ChesI’ in [12]). Representatives of this lineage were recently isolated from Kaneohe Bay [54], and a genome was sequenced from one strain (HIMB11; B. P. Durham et al., unpubl.). Several features distinguish this lineage from other characterized members of the Roseobacter clade, including its obligate oligotrophic nature, small genome size, small cell size, and atypical transporter repertoire. The cultivated strains and associated genome sequence are useful tools to help decipher the environmental factors and metabolic features responsible for bloom formation of this lineage.

In contrast to the marine Roseobacter clade, the betaproteobacterial lineage that increased in abundance immediately after the storm event was rare during non-storm conditions. Phylogenetic analyses placed this lineage within the bacterial family *Comamonadaceae*, where it appeared to be most closely related to a marine oligotroph isolated from the Baltic Sea [55], environmental gene clones recovered from seawater associated with mangroves in Taiwan [56] and brackish water of a southwestern Atlantic Ocean coastal lagoon [57], and members of the genus *Hydrogenophaga* [58] (Fig. S4). While most characterized members of the genus *Hydrogenophaga* have been isolated from highly eutrophic environments such as wastewater and activated sludge, strain BAL58 is an obligately oligotrophic marine bacterium isolated by extinction culture [55]. Elucidation of the specific environmental drivers and corresponding physiological traits that are responsible for the post-storm bloom of this lineage must await additional study.

While the two bacterial lineages considered above drove the statistically significant response to storm conditions at stations AR and NB, they did not display similar dynamics at stations CB and SB, which also exhibited statistically different bacterioplankton community structures between storm and non-storm conditions. When all four stations were considered, other bacterial groups were also found to be important drivers of the microbial community succession immediately after the storm event. Two of these groups, subclades of the SAR11 clade and members of the marine cyanobacterial genus *Synechococcus*, are abundant, ubiquitous members of the bacterioplankton community in this subtropical embayment during both storm and non-storm conditions. Thus, the rapid recovery of bacterioplankton community assemblages to pre-storm conditions appears due to a combination of rapid bloom and bust dynamics of otherwise relatively rare members of the bacterial community, as well as fluctuations in the abundance of bacteria that are already prevalent across the system.

Despite high spatial and temporal variability, we have shown that microbial (<1.6 µm) community composition and dynamics are linked to abiotic environmental forcing in this coastal subtropical environment. When coupled with the complex physical characteristics of this coastal system, the highly episodic and localized nature of storm-induced perturbation results in significant differences in the magnitude and character of disturbance experienced by different locations throughout the embayment. Characteristics of community composition itself, specifically the regional species pool (metacommunity) and resistance of the local (site-specific) community, appear to play important roles in microbial community responses to disturbances and the rapid recovery of this system from a major environmental forcing event.

## Supporting Information

Figure S1 **Contour plots showing spatial variation in median values of chlorophyll** ***a*** **concentrations, non-pigmented, prokaryotic cell abundances, N+N concentrations, and SRP concentrations in surface waters of Kaneohe Bay during non-storm (top panels) and storm and immediate post-storm (March 3–12, 2006; bottom panels) conditions. Filled circles represent sampling sites.**
(TIF)

Figure S2 **Two dimensional non-metric multidimensional scaling plots of SSU rRNA gene T-RFLP profiles using Bray-Curtis similarity for all stations sampled during non-storm (top panel) and storm (bottom panel; March 3 – 12, 2006) conditions.** Symbols represent individual sampling stations. Dashed lines indicate samples that share 60% similarity, while solid lines indicate 40% similarity.
(EPS)

Figure S3 **Two dimensional non-metric multidimensional scaling plots of SSU rRNA gene T-RFLP profiles using Bray-Curtis similarity for three of the four stations that had significant differences between storm and non-storm microbial community composition.** The fourth station (NB) is shown in Figure 3.
(EPS)

Figure S4 **Phylogenetic relationships between SSU rRNA gene clone sequences obtained from Station NB in Kaneohe Bay (prefixes NB031206 and NB062606) and representatives of the Family** ***Comamonadaceae*** **of the** ***Betaproteobacteria*** **.** The scale bar corresponds to 0.05 substitutions per nucleotide position. Open circles indicate nodes with bootstrap support between 50–80%, while closed circles indicate bootstrap support >80%, from 450 replicates. A variety of *Gammaproteobacteria* were used as outgroups (not shown).

Table S1 **Coordinates of stations in Kaneohe Bay, Hawaii, sampled in this study.**
(PDF)

Table S2 **Summary of 16S rRNA gene clones recovered from station NB during storm and non-storm conditions.**
(PDF)
