# Supplementary figures and images for: Coastal Bacterioplankton Community Dynamics in Response to a Natural Disturbance
Source: PLoS One. 2013 Feb 7;8(2):e56207. doi: 10.1371/journal.pone.0056207 (PMC3567041; doi:10.1371/journal.pone.0056207)

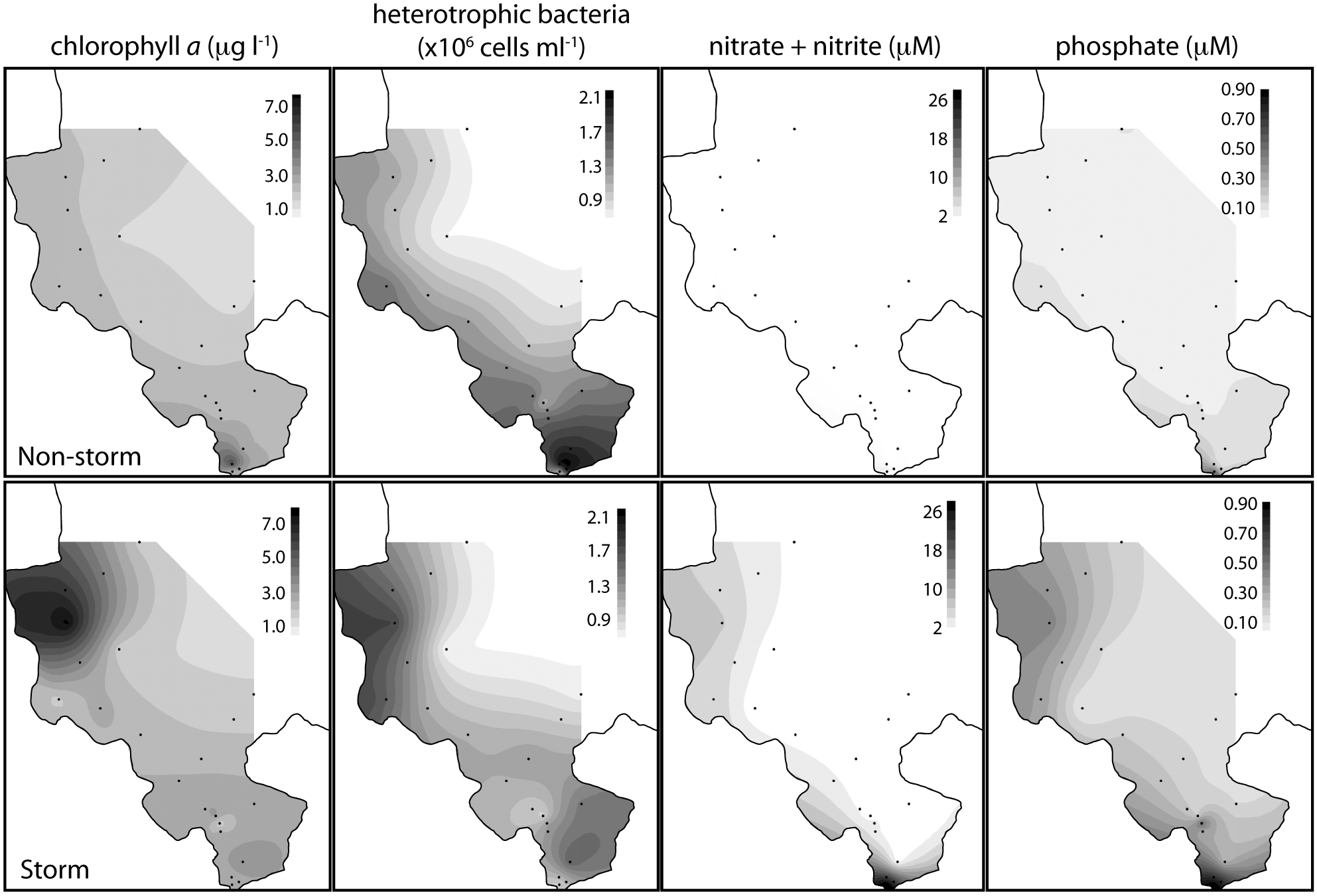

Supplement: Figure S1 — Contour plots showing spatial variation in median values of chlorophyll a concentrations, non-pigmented, prokaryotic cell abundances, N+N concentrations, and SRP concentrations in surface waters of Kaneohe Bay during non-storm (top panels) and storm and immediate post-storm (March 3–12, 2006; bottom panels) conditions. Filled circles represent sampling sites. (TIF) [file pone.0056207.s001.tif]
