# Supplementary material for: Coastal Bacterioplankton Community Dynamics in Response to a Natural Disturbance
Source: PLoS One. 2013 Feb 7;8(2):e56207. doi: 10.1371/journal.pone.0056207 (PMC3567041; doi:10.1371/journal.pone.0056207)
Supplement: Table S1 — Coordinates of stations in Kaneohe Bay, Hawaii, sampled in this study. (PDF) [file pone.0056207.s005.pdf]

Table S1. Coordinates of stations in Kaneohe Bay, Hawaii sampled in this study

Stations	Latitude (all 21º N)	Longitude (all 157º W)
AR	28.059’	50.151’
CB	27.423’	48.681’
CBC	28.721’	49.771’
CBS	28.956’	49.067’
JB	27.898’	49.067’
JD1	24.636’	49.406’
JD2	24.865’	47.352’
JD3	24.781’	47.047’
KS	24.730’	46.918’
MMRP	25.966’	47.042’
MR	26.596’	47.333’
NB	29.429’	49.997’
NBD	30.020’	50.034’
NR2	30.882’	48.703’
NR4	30.320’	49.350’
SB	26.181’	46.642’
SBC	25.685’	47.245’
SBE	25.141’	46.847’
SISLE	25.833’	47.263’
SR2	28.150’	46.651’
SR4	27.699’	47.010’
SR8	26.990’	47.593’
SY	26.088’	47.525’
