# Supplementary material for: Coastal Bacterioplankton Community Dynamics in Response to a Natural Disturbance
Source: PLoS One. 2013 Feb 7;8(2):e56207. doi: 10.1371/journal.pone.0056207 (PMC3567041; doi:10.1371/journal.pone.0056207)
Supplement: Table S2 — Summary of 16S rRNA gene clones recovered from station NB during storm and non-storm conditions. (PDF) [file pone.0056207.s006.pdf]

Table S2. Summary of 16S rRNA gene clones recovered from station NB during storm and non-storm conditions

Phylogenetic affiliation	Closest relative (Accession no.)	Storm[a]	Non-storm[b]	T-RF[c]	Representative clones
Alphaproteobacteria					
Rhodobacterales					
Unclassif. *Rhodobacteraceae*	Clone CB22G04, Chesapeake Bay, surface (EF471521)	64	31	34	NB031206_74, NB031206_113, NB031206_148, NB031206_167, NB031206_174, NB062806_208, NB062806_301
Phaeobacter/*Leisingera*	*Leisingera aquimarina* (AM900415)	5	-	34	NB031206_14
Unclassif. *Rhodobacteraceae*	Clone CB11B12, Chesapeake Bay, surface (EF471453)	1	3	34	NB062806_265
Pelagicola	*Pelagicola litoralis* (EF192392)	1	1	34	NB062806_278
Loktanella	*Loktanella hongkongensis* (AY600300)	2	-		NB031206_103
Unclassif. *Rhodobacteraceae*	Clone IBEA_CTG_2112418, Sargasso Sea (AACY01072860)	3	1	34	NB031206_17, NB031206_64, NB031206_83, NB062806_215
Unclassif. *Rhodobacteraceae*	Clone IBEA_CTG_2000148, Sargasso Sea (AACY01008274.1)	-	1		NB062806_242
Rickettsiales					
SAR11 subgroup IA	Clone Chl1.47, Gulf Stream, chlorophyll max. (DQ071059)	10	3	113	NB031206_32, NB031206_62, NB031206_93, NB031206_186, NB031206_133, NB031206_171, NB031206_94, NB031206_95, NB062806_193, NB062806_285, NB062806_317
SAR11 subgroup II	Clone 6C232648, tropical Pacific Ocean, surface (EU804691)	1	2	290	NB031206_22, NB062806_216, NB062806_267
SAR11 subgroup III	Clone CB11E07, Chesapeake Bay, surface (EF471461)	-	2	290	NB062806_198, NB062806_270
SAR11 subgroup V	Clone AEGEAN_169, Aegean Sea, 200 m (AF406519)	-	1	61	NB062806_218
Rhodospirillales					

SAR116 subgroup II	Clone OCS24, Oregon Coast, surface (AF001637)	1	5	225	NB031206_154, NB062806_237, NB062806_241, NB062806_247, NB062806_252, NB062806_352
SAR116 subgroup III	Clone 1C226889, Newport Harbor, RI, surface (EU799307)	1	1		NB031206_121
SAR116 subgroup IV	Clone CB22C04, Chesapeake Bay, surface (EF471706)	-	2		NB062806_209, NB062806_353
SAR116 subgroup I	Clone HF70_D1_P2, Station ALOHA, 70 m (DQ300819)	-	1		NB062806_308
Kiloniellales					
Kiloniella	Endosymbiont of scallop *Chlamys farreri* (AY174895)	-	1	34	NB062806_283
Unclassif. *Alphaproteobacteria*					
Pseudovibrio	*Pseudovibrio denitrificans* (AY486423)	1	-		NB031206_180
Gammaproteobacteria					
Alteromonadales					
Alteromonas	Clone M26-022, coral mucus enrichment (AM941178)	5	-	34	NB031206_107
Thalassomonas	*Thalassomonas loyana* (AY643537)	1	-		NB031206_87
Oceanospirillales					
OM252	Clone CHAB-III-7, coastal Mediterranean Sea (AJ240921)	1	2	34	NB031206_189, NB062806_290, NB062806_361
Unclassif. *Gammaproteobacteria*					
OM60 subgroup III	Clone Reef_E22, coastal Caribbean Sea, surface (GU119188)	-	7	34	NB062806_287, NB062806_348, NB062806_357, NB062806_382
OM60 subgroup I	Clone SPOTSOCT00_5m87, coastal Pacific Ocean, surface (DQ009137)	3	2	34	NB031206_53, NB062806_249, NB062806_360
SAR86 subgroup I/II	Clone 1C226807, Newport Harbor, RI, surface (EU799230)	2	5	187	NB031206_120, NB062806_223, NB062806_321
SAR86 subgroup V	Clone 6C233101, tropical Pacific Ocean, surface (EU805114)	1	-	34	NB031206_43
PC-FL10-68	Clone PC-FL10-68, coastal Hong Kong, surface (EF379881)	3	3	187	NB031206_67, NB062806_201, NB062806_381

SAR145	Clone c24, Northern Bering Sea, bottom water (GQ452905)	4	-	207	NB031206_97, NB031206_165
OM182 subgroup I	Clone HF10_02O24, Station ALOHA, surface (EU361650)	-	1		NB062806_368
OM182 subgroup II	Clone SPOTSAPR01_5m247, coastal Pacific Ocean, surface (DQ009154)	1	-	187	NB031206_86
HTCC2151	Clone SHAB744, Saanich Inlet, surface (GQ348802)	-	1		NB062806_342
Unique	Clone d131, Northern Bering Sea bottom water (GQ850579)	4	-	65	NB031206_69
Unique	Clone SGUS989, coral-associated (FJ202922)	1	-		NB031206_143
Unique	Clone ctg_NISA227, deep-sea octacoral (DQ396123)	1	-		NB031206_166
Unique	Clone 1C, clam gill symbiont (FJ752446)	-	1		NB062806_355
Betaproteobacteria					
Burkholderiales					
Uncultured *Comamonadacae*	Clone RS62, Atlantic Ocean lagoon, surface (DQ450189)	22	-	197	NB031206_13, NB031206_19, NB031206_68, NB031206_75
Methylophilales					
OM43	Strain HIMB624, coastal Hawaii, surface (EU433381)	6	11	221	NB031206_55, NB031206_182, NB062806_228, NB062806_282
Unclassif. *Betaproteobacteria*					
Unique	Clone Reef_P05, coastal Caribbean Sea, surface (GU119402)	3	-	200	NB031206_9, NB031206_54
Deltaproteobacteria					
Myxococcales					
Unique	Clone HF10_F9_P1, Station ALOHA, surface (DQ300652)	-	2		NB062806_227, NB062806_369
Unclassif. *Deltaproteobacteria*					
OM27	Clone SHAB701, Saanich Inlet, surface (GQ348771)	5	1		NB031206_100, NB031206_115, NB031206_136, NB031206_158

Unique	Clone Reef_A22, coastal Caribbean Sea, surface (GU119488)	-	3		NB062806_264, NB062806_325, NB062806_347
Unique	Clone IBEA_CTG_2013058, Sargasso Sea (AACY01079896.1)	1	-		NB031206_89
Bacteriodetes					
Flavobacteriales					
Unclassif. *Flavobacteriales*	Clone M0-sP3C09, coastal Pacific Ocean (EF016485)	-	10	34	NB062806_280, NB062806_327, NB062806_350
Unclassif. *Flavobacteriales*	Clone Reef_M01, coastal Caribbean Sea, surface (GU119263)	2	10	34	NB031206_176, NB062806_196, NB062806_211, NB062806_277
Mesoflavibacter	Clone SCRIPPS_413, dinoflagellate culture (AF359548)	1	-		NB031206_145
Unclassif. *Flavobacteriaceae*	Clone SGSO744, Saanich Inlet, surface (GQ347950)	1	-		NB031206_141
Unclassif. *Cryomorphaceae*	Clone Reef_B06, coastal Caribbean Sea, surface (GU119331)	-	1		NB062806_199
ZD0403	Clone Reef_N17, coastal Caribbean Sea, surface (GU119256)	1	-		NB031206_163
Unclassif. *Flavobacteriaceae*	Clone MAW17, Mediterranean Sea marina (AM747353)	-	2		NB062806_197
Unclassif. *Flavobacteriaceae*	Clone SHAB680, Saanich Inlet, surface (GQ348753)	-	1		NB062806_316
Unclassif. *Flavobacteriaceae*	Clone M0-Ar2-P4A07, coastal Pacific Ocean (EF016474)	-	1		NB062806_204
Sphingobacteriales					
Unclassif. *Saprospiraceae*	Clone agg32, marine snow (L10944)	-	1		NB062806_341
Unclassif. *Sphingobacteriales*	Clone CB11D01, Chesapeake Bay, surface (EF471578)	-	1		NB062806_366
Planctomycetes					
Unclassif. *Planctomycetaceae*	Fosmid clone 6FN, Namibian Shelf, 52 m (EF591887)	-	1		NB062806_377
Actinobacteria					
Marine *Actinobacteria* clade	Clone A313034, Arabian Sea, 50 m	1	5	327	NB031206_152, NB062806_288

	(AY907743)				
Cyanobacteria					
Chloroplast					
Unclassif. *Prasinophyceae*	Clone AEGEAN_115, Aegean Sea, 200 m (AF406549)	7	5	383, 384	NB031206_29, NB031206_46, NB031206_159, NB062806_220, NB062806_226
Unclassif. *Prasinophyceae*	Clone OM5, coastal Atlantic Ocean, surface (U70715)	3	-		NB031206_104
Bacillariophyta	*Nitzschia frustulum* chloroplast (AY221721)	-	2	377	NB062806_224, NB062806_293
Bacillariophyta	*Thalassiosira pseudonana* chloroplast (EF067921)	-	1		NB062806_214
Bacillariophyta	Clone SGSO524, Saanich Inlet, surface (GQ347794)	-	3	377	NB062806_213, NB062806_261
Synechococcus					
Synechococcus clade II	*Synechococcus* sp. RS9912 (AY172822)	1	18	289	NB062806_200, NB062806_383
Synechococcus clade V-VII	*Synechococcus* sp. RS9920 (AY172830)	-	1		NB062806_338, NB062806_363
Synechococcus clade IX	*Synechococcus* sp. RS9901 (AY172811)	1	6	289	NB031206_78, NB062806_306
Unique	*Synechococcus* sp. RS9915 (AY172825)	-	17	135	NB062806_240, NB062806_351
Unique	Clone CB11C04, Chesapeake Bay, surface (EF471456)	-	2		NB062806_253
Firmicutes					
Clostridiales					
Fusibacter	Clone SGUS647, coral-associated (FJ202768)	2	-		NB031206_25, NB031206_26
Unclassif. *Clostridiales*	Clone RB_13f, coral-associated (EF123527)	1	-		NB031206_192

a. Storm sampled March 12, 2006; clone prefix “NB031206”; n=175
b. Non-storm sampled June 28, 2006; clone prefix “NB062806”; n=182
c. Actual length of the T-RF (in base pairs), determined by T-RFLP analysis of representative clones, is indicated
